# Oncogenic EGFR Signaling as a Central Regulator of Chemoresistance in Ovarian Cancer: A Mechanistic Review

**DOI:** 10.3390/ijms27135937

**Published:** 2026-07-01

**Authors:** Arulkumar Nagappan, Veeran Sethuraman, Parthiban Pandian, Jothi Nedunchezhian, Arvind Kumar Shukla

**Affiliations:** 1 Department of Pathology, Saveetha Medical College and Hospital, Saveetha Institute of Medical and Technical Sciences (SIMATS), Saveetha University, Thandalam, Chennai 602105, Tamil Nadu, India; dr.parthi2009@gmail.com; 2 Department of Orthopaedics, Saveetha Medical College and Hospital, Saveetha Institute of Medical and Technical Sciences (SIMATS), Chennai 602105, Tamil Nadu, India; sethuramanbio@gmail.com; 3 PG & Research Department of Biotechnology & Bioinformatics, Holy Cross College (Autonomous), Affiliated to Bharathidasan University, Tiruchirappalli 620002, Tamil Nadu, India; jothi@hcctrichy.ac.in; 4 School of Biomedical Convergence Engineering, Pusan National University, Yangsan 50612, Republic of Korea

**Keywords:** ovarian cancer, EGFR, chemoresistance, targeted therapy, tumor microenvironment, EMT, cancer stem cells

## Abstract

Ovarian cancer (OVC) is a leading cause of gynecological cancer mortality due to late-stage diagnosis and chemoresistance. Among the multiple molecular mediators, oncogenic epidermal growth factor receptor (EGFR) signaling has emerged as a key regulator of tumor progression and drug resistance, ultimately governing cancer survival. Therefore, this review focused on the molecular mechanisms of aberrant EGFR signaling to promote chemoresistance in ovarian cancer through multiple interlinking pathways, including the phosphoinositide 3-kinase (PI3K)/protein kinase B (AKT)/mammalian target of the rapamycin (mTOR), mitogen-activated protein kinase (MAPK)/extracellular signal-regulated kinase (ERK), and Janus kinase (JAK)/signal transducer and activator of transcription (STAT) signaling cascades. These pathways act in concert to confer resistance, including proliferation, antiapoptotic effects, cancer stem cell maintenance, and facilitating epithelial-mesenchymal transition (EMT), which function together to decrease sensitivity towards platinum-based and taxane chemotherapies. Furthermore, we incorporate novel evidence regarding EGFR cross-talk with extracellular matrix (ECM) and metabolic reprogramming, especially their relevance to immune evasion mechanisms, hypoxia, and extracellular vesicles (EVs)-mediated signaling. In addition, we elaborated on the limitation of the current EGFR targeting therapy, which will be beneficial for further designing new combinatorial treatment approaches by using EGFR inhibitors with immunotherapy, nanocarriers, and microbiota modulators. Overall, this review highlights the updated role of EGFR signaling as a key regulator of chemoresistance in ovarian cancer, providing insights for developing targeted therapies to overcome drug resistance and improve patient survival.

## 1. Introduction

Ovarian cancer (OVC) is one of the most lethal forms of gynecological cancer, as it causes a great public health concern in terms of its advanced stage of detection, tendency to recur, and minimal success in achieving positive therapeutic results [1,2]. Epidemiologically, epithelial ovarian cancer (EOC) accounts for approximately 90% of all ovarian cancer cases and is frequently diagnosed at an advanced stage, contributing to poor survival outcomes [3]. Despite the initial sensitivity to the therapy, the overall five-year survival rate is still quite low [4]. The optimal treatment options for ovarian cancer involve cytoreductive surgery and then the use of platinum-containing chemotherapy, normally combined with taxanes. Although more than 70% of patients respond positively to platinum-based therapies initially, many will go on to relapse and become resistant, becoming the major reason behind mortality in such cases [5]. This resistance may arise due to different factors and pathways, such as increased ability to repair damaged DNA, drug efflux, EMT, or changes in the tumor microenvironment (TME) [6,7]. As a result, drug resistance has emerged as one of the major problems in managing ovarian cancer.

According to emerging data, chemoresistance appears to result from a sophisticated process of inter-connected signaling networks where receptor tyrosine kinases (RTKs), such as epidermal growth factor receptor (EGFR), have been found to exert critical roles [8,9]. The expression of EGFR is frequent in ovarian cancer, ranging from 30% to 98% in patients [10,11] and correlating with the progression of the disease as well as resistance to chemotherapy. Activating EGFR initiates several signaling pathways, e.g., phosphoinositide 3-kinase (PI3K)/protein kinase B (AKT)/and rat sarcoma RAS/mitogen-activated protein kinase (MAPK) pathway, responsible for cellular proliferation, survival, migration, and apoptosis [10]. Dysfunction of the above-mentioned processes through deregulating these two pathways is a direct cause of chemoresistance through suppressing apoptosis and enhancing tumor survival [12,13]. In addition, numerous investigations have established a relationship between activation of the EGFR signaling network and resistance to platinum-based chemotherapy [14,15]. The latter includes a positive association between the activity of EGFR and decreased sensitivity to cytotoxic drugs, while in turn, EGFR pathway inhibition increases the susceptibility to the treatment [10]. Moreover, EGFR cooperates with the other oncogenic factors, including those in the tumor microenvironment, contributing to chemoresistance regulation [16].

Given the wide dysregulation of its functional significance, as well as druggability, it is clear that EGFR signaling is a very interesting therapeutic target in ovarian cancer. Despite these advances, the clinical efficacy of EGFR-targeted therapies remains limited, highlighting the need for a deeper understanding of the complex molecular networks that drive EGFR-associated therapeutic resistance and for more precise patient stratification approaches. Hence, this review attempts to focus on the mechanisms underlying oncogenic EGFR-mediated resistance to chemotherapy in order to develop effective targeted therapy.

## 2. EGFR Signaling Pathway: Molecular Architecture and Activation

**Figure 1:**
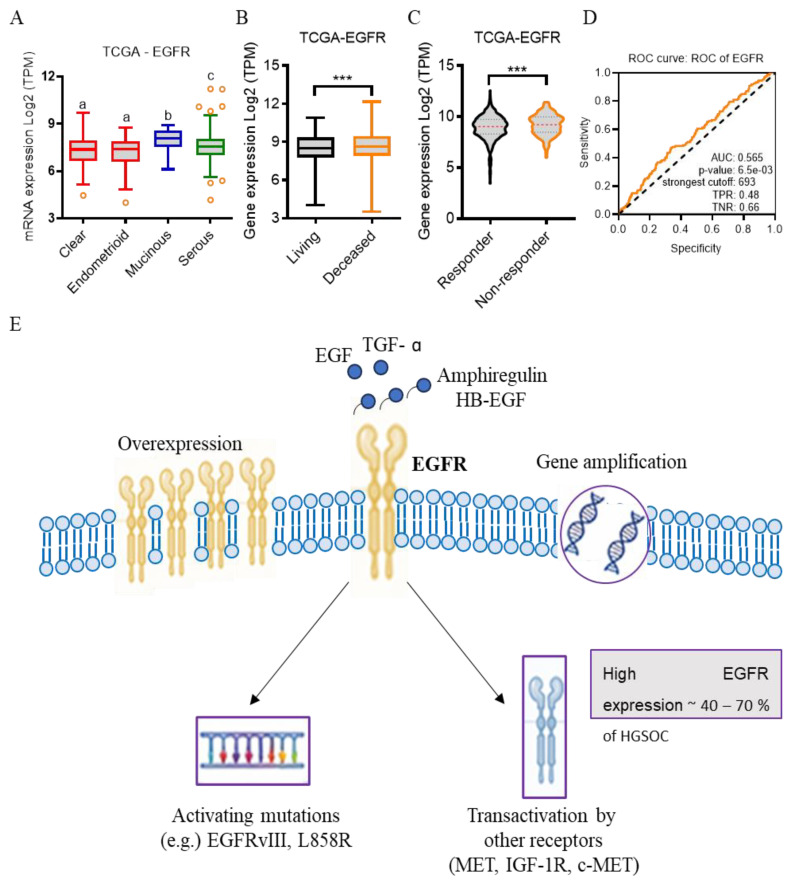
EGFR signaling is frequently activated in ovarian cancer and correlates with poor response to chemotherapy. (**A**) EGFR expression in histological subtypes of ovarian cancer stages was derived from the TCGA-OVC (*n* = 940) dataset (http://gent2.appex.kr/gent2/, accessed on 29 April 2026). The results were shown as a box-whisker plot. Different letters over each bar represent significant differences between groups (*p* < 0.05). (**B**) Overall survival (OS) status of patients with low and high expression levels of EGFR from The Cancer Genome Atlas-ovarian serous cystadenocarcinoma (TCGA-OSC, *n* = 617), Firehose Legacy dataset (https://www.cbioportal.org/, accessed on 29 April 2026). The results were shown as a box-whisker plot. The asterisks (*) indicate significant differences between the two groups (*** *p* < 0.001). (**C**,**D**) ROC curve for EGFR gene (Affymetrix ID: 201983_s_at/EGFR) of pathological responses to chemotherapeutics (Platin & Taxane) in ovarian cancer patients (*n* = 834) (https://rocplot.com/, accessed on 29 April 2026). The results were shown as a violin plot. The asterisks (*) indicate significant differences between the two groups (*** *p* < 0.001). (**E**) Mechanisms of EGFR activation in ovarian cancer. EGFR can be activated through multiple mechanisms, including ligand binding (e.g., EGF, TGF-α, amphiregulin, HB-EGF), receptor overexpression, gene amplification, activating mutations (e.g., EGFRvIII, L858R), and transactivation by other receptors such as MET, IGF-1R, and c-MET. Ultimately, these events stimulate downstream signaling pathways that promote cancer. EGFR, epidermal growth factor receptor; EGF, epidermal growth factor; OVC, ovarian cancer; ROC, receiver operator characteristic; TCGA, The Cancer Genome Atlas; TGF-α, transforming growth factor-α; HB-EGF, heparin-binding EGF-like growth factor; IGF-1R, insulin-like growth factor 1 receptor; c-MET, cellular mesenchymal-epithelial transition factor; HGSOC, high-grade serous ovarian cancer.

The Cancer Genome Atlas (TCGA) ovarian cancer dataset showed differential expression of EGFR mRNA across histological subtypes, suggesting EGFR expression may be different depending on the ovarian cancer histology and may contribute differently to tumor progression and therapeutic response (Figure 1A). Survival-associated analysis of TCGA ovarian cancer samples revealed that EGFR expression was significantly elevated in deceased patients compared with living patients (*** *p* < 0.001), revealing a potential association between EGFR overexpression and poor clinical outcome (Figure 1B). Furthermore, the TCGA dataset revealed higher EGFR expression in non-responders than in responders (Figure 1C). A receiver operator characteristic (ROC) analysis of EGFR might be useful in predicting ovarian cancer prognosis (Figure 1D).

### 2.1. Structure and Ligand Binding of EGFR

EGFR is a transmembrane receptor tyrosine kinase of the ErbB family. It is comprised of an extracellular ligand-binding domain, a single transmembrane helix, and an intracellular tyrosine kinase domain. Ligand binding (usually EGF, TGF-α, or amphiregulin) induces receptor dimerization (homodimerization or heterodimerization with HER family members), which causes autophosphorylation of specific tyrosine residues within the cytoplasmic domain (Figure 1E) [17,18]. These phosphorylated residues then act as docking sites for adaptor proteins, such as GRB2 and SHC, to initiate multiple downstream signaling cascades [9,19]. In ovarian cancer, EGFR is typically overexpressed with wild-type sequence [20]. Ligand-independent activation through receptor cross-talk or transactivation can further increase EGFR signaling to promote tumor cell proliferation and survival [21,22].

### 2.2. Canonical Downstream Pathways

Therapeutic strategies targeting this receptor have been developed and include the use of monoclonal antibodies (cetuximab, panitumumab), or small molecule kinase inhibitors (erlotinib, gefitinib). Indeed, EGFR is a validated target in the treatment of metastatic colorectal cancer and head and neck cancers, highlighting its relevance as a therapeutic target [16,23,24]. Therefore, pathological activation or overexpression of EGFR gene is a common hallmark in solid tumors. The EGFR is a receptor tyrosine kinase often overexpressed or aberrantly activated in ovarian cancer. EGFR initiates multiple intracellular signaling cascades promoting tumor progression and chemoresistance (Table 1) [5,25,26,27,28]. Upon ligand binding or oncogenic activation, EGFR dimerizes and autophosphorylates, leading to recruitment of adaptor proteins to initiate downstream signaling cascades PI3K/AKT/mammalian target of rapamycin (mTOR), mitogen-activated protein kinase (MAPK)/extracellular signal-regulated kinase (ERK), and Janus kinase (JAK)/signal transducer and activator of transcription (STAT) and phospholipase C-γ (PLC γ)/protein kinase C (PKC) among others that ultimately regulate proliferation, survival, metabolism and stress adaptation [29,30,31].

#### 2.2.1. PI3K/AKT/mTOR Pathway

One of the most important targets for EGFR signaling is the PI3K/AKT/mTOR axis. When EGFR is phosphorylated, PI3K is recruited to it, resulting in the production of PIP3 and thus activation of AKT. Once activated, AKT promotes cell survival by blocking pro-apoptotic proteins and promoting protein synthesis through the mTOR pathway. In patients with ovarian cancer, the PI3K/AKT/mTOR axis is often dysregulated through either a mutation in PIK3CA or a loss of PTEN, resulting in constant activation of this pathway [5,27,28,36,37,38]. By preventing apoptosis, enhancing DNA repair, and enhancing progress through the cell cycle, the PI3K/AKT pathway is a significant contributor to the chemotherapy resistance observed in patients [39]. For example, AKT activation has been associated with chemotherapy resistance to platinum-based chemotherapy by inhibiting apoptotic pathways and increasing cell survival [29].

#### 2.2.2. RAS/RAF/MEK/ERK Pathway

The MAPK pathway is another major signaling cascade activated by EGFR. Upon receptor activation, the function of adaptor proteins can trigger the activation of RAS that leads to the activation of a kinase cascade involving RAF, MEK, and ERK [30,31,40]. The activated form of ERK moves into the nucleus where it regulates gene expression related to proliferation and differentiation [41,42]. This pathway is commonly found abnormally activated in ovarian cancer, particularly in the low-grade serous subtype; this can be demonstrated by the frequent occurrence of KRAS and BRAF mutations [43,44]. Sustained activation of ERK has been shown to promote chemoresistance through increased cellular proliferation, decreased apoptosis, and the facilitation of EMT [33,34]. Furthermore, hyperactivation of the MAPK pathway is strongly associated with resistance to platinum-based therapies [12,45].

#### 2.2.3. JAK/STAT Pathway

EGFR activation influences the JAK/STAT pathway, specifically STAT5 and STAT3 activation. Following activation, STAT proteins form dimers and move into the nucleus, regulating genes associated with proliferation, immune evasion, and survival [46,47]. Continuous activation of the STAT pathway leads to resistance to chemotherapy via the upregulation of anti-apoptotic proteins (e.g., Bcl-xL), the production of inflammatory signals in the tumor microenvironment, and a greater chance of tumor cell survival through the interaction of the EGFR and STAT pathways, which contributes to resistance to chemotherapy in ovarian carcinoma [5,29,32].

### 2.3. Signal Integration and Pathway Cross-Talk

EGFR signaling in ovarian cancer is distinguished from other malignancies by unique levels of integration within the greater oncogenic signaling network. This allows tumors to escape the effects of targeted therapies and create an environment permitting continued growth despite exposure to anticancer treatments. One mechanism used by tumors to promote their continued growth is the activation of compensatory (or parallel) signaling circuitries. For example, the simultaneous stimulation of PI3K/AKT/mTOR pathway or IGF signaling often blocks EGFR inhibition from having any therapeutic impact [36]. Additionally, through the cross-talk of PI3K/AKT pathway and MAPK pathway, cross-talk can cause a reciprocal activation of downstream signaling pathways, even if one of the pathways is inhibited.

Additionally, the PI3K/AKT and MAPK pathways both enable the phosphorylation and inactivity of pro-apoptotic proteins (like BAD), facilitating cell growth/survival and conferring some degree of resistance to chemotherapy [12]. Such redundancy illustrates the difficulty of targeting one specific pathway in ovarian cancer. Also, more recent data indicate that miRNAs and other regulatory molecules may modulate EGFR signaling networks in order to facilitate this adaptiveness. For instance, alterations in miRNA expression can also regulate important components of both the MAPK and PI3K/AKT pathways, which may contribute to chemoresistance for the cancerous cells of the ovary [10]. Many of the cytokines and growth factors found in the tumor microenvironment have also been implicated in the indirect activation of EGFR signaling, which would contribute to the resistance mechanisms [29]. High levels of G-protein-coupled receptor (GPCR) activity have also been shown to transactivate (activate through another pathway) EGFR, adding even more diversity to the available EGFR signaling cascades [29].

## 3. Dysregulation of EGFR in Ovarian Cancer

The EGFR, part of the ErbB family of receptor tyrosine kinases, plays an essential role in the regulation of cell proliferation, differentiation, survival, and migration. In ovarian cancer, aberrant signaling of EGFR represents a major molecular event contributing to tumor development, progression, and treatment resistance [48,49]. Dysregulation of EGFR occurs via multiple mechanisms, including both overexpression as well as gene amplification, mutation and ligand-dependent hyperactivation. These dysregulations ultimately result in sustained activation of oncogenic downstream pathways (e.g., PI3K/AKT, MAPK/ERK, JAK/STAT), giving rise to a survival advantage for ovarian cancer cells that are exposed to treatment stressors [8,50].

### 3.1. EGFR Overexpression and Amplification

Approximately 30% to 70% of EOC patients show evidence of overexpression of EGFR; EGFR overexpression represents one of the most common alterations seen in patients with EOC [10,11,51]. Additionally, EGFR overexpression often results from amplification of the EGFR gene, which leads to increased receptor density on the surface of cells, as well as increased sensitivity of the receptors to their ligands. As a result, even very low concentrations of ligands (e.g., EGF or TGF-α) can result in sustained stimulation of the receptors [52]. In general, EGFR overexpression increases activation of anti-apoptotic and pro-survival (proliferation) pathways, particularly PI3K/AKT signaling, which inhibits pro-apoptotic factors [e.g., BAD] and promotes the survival of cells [53,54]. Furthermore, stimulation of MAPK/ERK signaling results in increased proliferation of cells and the overall growth of tumors; collectively, these pathways are believed to contribute to the development of resistance to platinum-based chemotherapy agents (e.g., cisplatin and carboplatin) by decreasing apoptosis and enhancing the ability of cells to repair DNA [50]. Finally, amplification of the EGFR gene is also associated with more aggressive tumors (i.e., more invasive and metastatic tumors), and studies have shown that EOC patients with amplification of EGFR have a poorer response to chemotherapy and shorter PFS than other patients with EOC [55,56]. EGFR overexpression has also been shown to be associated with increased expression of drug efflux transporters (e.g., ABCB1), thereby contributing to multidrug resistance [12,30,35,57].

### 3.2. Mutations and Mutation-Mediated Aberrant Activation

While EGFR mutations are commonly seen in non-small cell lung cancer, the presence of mutated EGFRs in ovarian cancer is less frequent. However, the activation of EGFR signaling through various means, including auto- and paracrine ligand synthesis, receptor dimerization with other ERBB receptors (e.g., HER2/ERBB2), and failure of degradation of EGFR, is frequently seen [58,59]. The formation of auto- or paracrine signaling “loops” by EGF and TGF-α result in constant activation of the receptor regardless of the presence or absence of external stimuli. Furthermore, the cross-talk between EGFR and other oncogenic pathways, such as JAK/STAT and NF-κB pathways, greatly enhances EGFR-mediated signaling [29]. Together, these pathways lead to increased expression of genes related to cell proliferation, angiogenesis and survival.

Another significant mechanism of abnormal activation of EGFR is the inability to carry out endocytosis of EGFR and degrade its proteins within cells. When active, EGFR can be taken up into the cell and degraded by lysosomes; however, this process is frequently disrupted in ovarian cancer, resulting in prolonged signaling from the receptor [60,61]. In addition, mutations of or dysregulation of negative regulators of EGFR, including PTEN, lead to increased activation of PI3K/AKT through EGFR-mediated signaling pathways. Sustained signaling is substantially responsible for chemoresistance among CRC cells. For example, EGFR-mediated activation enhances DNA repair pathways and decreases the effectiveness of platinum-based chemotherapeutic agents [62]. Additionally, EGFR activation causes HNSCC cells to undergo epithelial to mesenchymal transition (EMT), a process characterized by increased migratory capability and apoptosis resistance [63,64]. EMT is caused by activation of Snail, Slug, and Twist, which are transcription factors that are activated by EGFR-mediated signaling.

### 3.3. Clinicopathological and Prognostic Significance

Multiple studies have explored the clinical implications of EGFR dysregulation in ovarian cancer with different degrees of assumptions about that relationship; however, many forms of evidence demonstrate the direct link between clinical outcome and EGFR overexpression, which was found to be linked with increased aggressiveness and late-stage disease. Patients with high EGFR levels are known to have lower overall and progression-free survival [65,66,67]. Additionally, EGFR dysregulation has been linked to resistance to first-line chemotherapy regimens, particularly platinum- and taxane-containing chemotherapies [68,69,70].

## 4. Mechanistic Basis of EGFR-Mediated Chemoresistance

**Figure 2:**
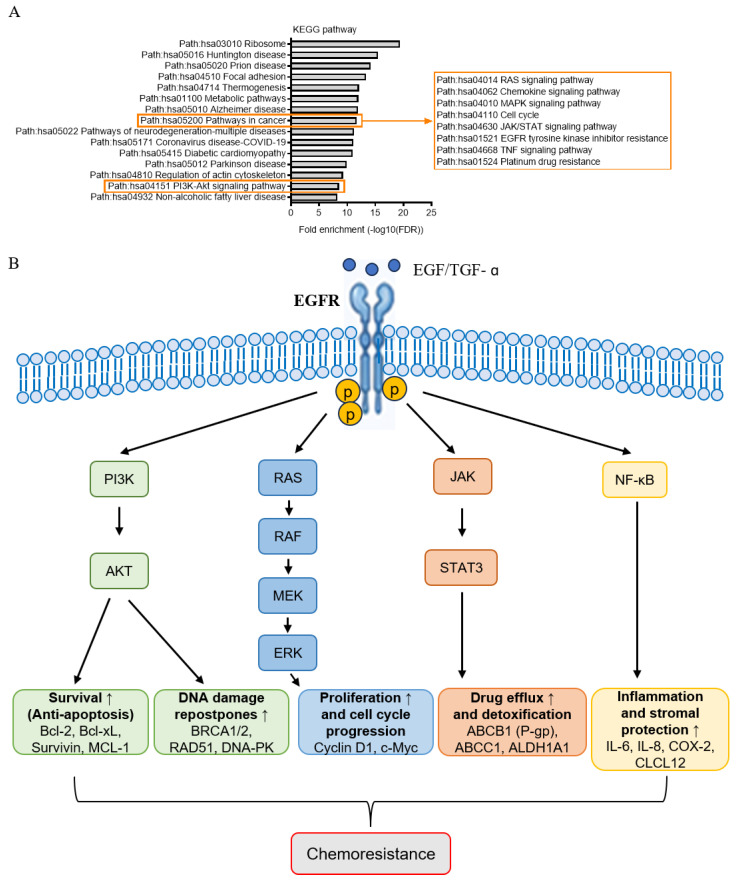
Oncogenic EGFR signaling drives multiple mechanisms of chemoresistance in ovarian cancer. (**A**) KEGG pathway enrichment for EGFR-positively co-expressed genes retrieved from The Cancer Genome Atlas-ovarian serous cystadenocarcinoma (TCGA-OSC, *n* = 617), Firehose Legacy dataset (https://www.cbioportal.org/, accessed on 29 April 2026). (**B**) Ligand-induced EGFR activation leads to receptor dimerization and autophosphorylation, triggering several downstream signaling cascades, including the PI3K/AKT pathway that promotes cell survival and inhibits apoptosis through upregulation of anti-apoptotic proteins (e.g., BCL-2, BCL-XL, Survivin); the RAS/RAF/MEK/ERK pathway enhances proliferation and cell cycle progression via regulators such as cyclin D1 and c-Myc; the JAK/STAT3 pathway increases drug efflux and detoxification through ABC transporters (e.g., ABCB1/P-gp, ABCC1) and ALDH1A1; the NF-κB pathway drives inflammatory signaling and stromal interactions (e.g., IL-6, IL-8, COX-2, CXCL12), contributing to tumor microenvironment remodeling. Collectively, these pathways lead to reduced apoptosis, enhanced DNA repair capacity, increased drug efflux, and tumor microenvironment alterations, ultimately resulting in chemoresistance. ABCB1 (P-gp), ATP-binding cassette subfamily B member 1 (P-glycoprotein); ABCC1, ATP-binding cassette subfamily C member 1; AKT, protein kinase B (PKB); ALDH1A1, aldehyde dehydrogenase 1 family member A1; Bcl-2, B-cell lymphoma 2; Bcl-xL, B-cell lymphoma-extra Large; BRCA1, breast cancer gene 1; BRCA2, breast cancer gene 2; c-Myc, cellular myelocytomatosis oncogene; COX-2, cyclooxygenase-2; CXCL12, C-X-C motif chemokine ligand 12; DNA-PK, DNA-dependent protein kinase; EGFR, epidermal growth factor receptor; ERK, extracellular signal-regulated kinase; IL-6, interleukin-6; IL-8, interleukin-8; JAK, Janus kinase; MCL-1, myeloid cell leukemia sequence 1; MEK, mitogen-activated protein kinase; NF-κB, nuclear factor kappa B; PI3K, phosphoinositide 3-kinase; RAD51, RAD51 recombinase; RAF, rapidly accelerated fibrosarcoma kinase; RAS, rat sarcoma; STAT3, signal transducer and activator of transcription 3; Survivin, baculoviral IAP repeat containing protein 5 (BIRC5). ↑—Upregulation; ↓—Downregulation.

To drive mechanistic insights into EGFR-positively co-expressed genes of ovarian cancer, KEGG enrichment analyses were performed. We found that EGFR correlated genes were mostly enriched in the pathways in cancer and the PI3K/AKT signaling pathway (Figure 2A). Additionally, bioinformatic analysis of the pathways in cancer also exhibited a notable enrichment in the multiple pathways, including RAS signaling, chemokine signaling, MAPK signaling, cell cycle, JAK/STAT signaling, and TNF signaling pathways, resistance to EGFR tyrosine kinase inhibitors, and platinum drugs, highlighting the potential role of EGFR-driven signaling networks in ovarian cancer progression and chemoresistance.

### 4.1. Activation of Pro-Survival Signaling

Activation of pro-survival signaling pathways (e.g., PI3K/AKT/mTOR and MAPK/ERK) via EGFR is the major mechanism through which EGFR provides a means for the chemosensitivity of ovarian cancer cells [38,71,72]. In chemoresistant ovarian cancer, the PI3K/AKT signaling axis is a major contributor to chemoresistance by increasing cell survival and proliferation and providing enhanced metabolic adaptability when chemotherapy is given [45]. In many cases, upregulation (activation) of these pathways results in a decreased sensitivity of ovarian cancer cells to chemotherapy, particularly in the case of platinum- and taxane-based therapies [6,14]. The activation of the PI3K/AKT axis, in particular, provides a survival signal to ovarian cancer cells by phosphorylating/inactivating pro-apoptotic proteins (e.g., BAD and caspase-9) and activating anti-apoptotic proteins (e.g., Survivin) [73,74]. Additionally, the MAPK signaling pathway contributes to chemoresistance by promoting progression and increased proliferation of the ovarian cancer cell cycle and enabling the tumor cells to develop resistance to the stress induced by the chemotherapeutic agent [5]. Furthermore, through the activation of NF-κB via EGFR, ovarian cancer cells may develop chemoresistance through the transcriptional activation of genes involved in the regulation of inflammation, survival, and drug efflux (Figure 2B). Collectively, the combination of the above pathways provides a durable pro-survival environment for ovarian cancer cells and allows them to evade chemotherapeutic-induced cell death. The molecular mechanisms and targets of EGFR signaling involved in chemoresistance in ovarian cancer are listed in Table 2.

### 4.2. Suppression of Apoptosis Pathways

Through the process of apoptosis, chemotherapy can destroy cancer cells. However, the action of the EGFR has an effect on apoptosis pathways and creates chemoresistant cancer cells. Chemoresistant ovarian cancer cells exhibit reduced apoptosis and are determined to have a decreased amount of expression of apoptotic regulators (BCL-2 family proteins) or the proteins that inhibit apoptosis (IAPs) [45,76]. When EGFR is activated, it increases the expression of anti-apoptotic proteins (i.e., BCL-2 and MCL-1) and reduces the expression of pro-apoptotic proteins (i.e., BAX and p53) [5,28]. Additionally, transcriptional factors that are associated with epithelial-mesenchymal transition (EMT), such as Snail and Slug, can inhibit p53-mediated apoptosis when activated by EGFR, thus providing an additional means for the survival of cancer cells [77]. The inhibition of apoptosis permits the cancer cell to become resistant to DNA damage induced by chemotherapy and thus creates the resistance mechanism for the cell (both intrinsic and acquired).

### 4.3. Enhancement of DNA Damage Repair

Chemotherapy drugs like cisplatin work by damaging the cellular DNA (especially through making DNA cross-links and causing double-strand breaks). However, EGFs can stimulate an enhanced response to DNA damage (also known as the DNA damage response, or DDR for short), which allows the cancer cell to repair its damaged DNA more efficiently, allowing the cancer cell to survive [78,79]. One specific mechanism for chemoresistance (the inability of a cell to respond to chemotherapeutics) in ovarian cancer is through an increased capacity of the cancer cell to repair its DNA [80]. Several major pathways responsible for DNA repair, such as homologous recombination (HR), nucleotide excision repair (NER), and base excision repair (BER), are all typically increased in tumors that show resistance to clinical chemotherapy [81,82]. EGFR signaling promotes this increased capacity for DNA repair by activating downstream proteins that regulate the expression levels of various DNA repair proteins (for example, BRCA1/2, RAD51, and PARP) [34,75]. In addition, several of the proteins that regulate the EMT process increase the levels of DNA repair enzymes (for instance, ERCC1 and PARP), thereby counteracting any cytotoxic effects that platinum-based chemotherapeutic agents may have on the tumor cells [77]. As a result of having an increased capacity for DNA repair, tumor cells are able to maintain their genomic integrity, even after being continually exposed to chemotherapeutic agents.

### 4.4. Induction of Epithelial-Mesenchymal Transition (EMT)

The activation of EGFR signaling creates a situation that favors the induction and development of epithelial-mesenchymal transition (EMT). An EMT is the point at which epithelial cells take on mesenchymal characteristics, such as increased motility and invasiveness, and resistance to apoptosis [83]. There is a strong link between chemoresistance and EMT in ovarian cancer. Epithelial markers (e.g., E-cadherin) are downregulated and mesenchymal markers (e.g., N-cadherin, vimentin, and fibronectin) are upregulated during the process of EMT [77,84]. The change in the phenotype of the tumor cells leads to enhanced aggressiveness and decreased sensitivity to chemotherapy drugs. Pathways involved in the activation of EGFR, such as PI3K/AKT and TGF-b/Smad, are key to regulating transcription factors that mediate EMT (Snail, Slug, Twist, ZEB1 and ZEB2) [85,86]. In addition, elevating levels of mesenchymal markers and transitioning from an epithelial to a mesenchymal phenotype lead to many mechanisms of resistance (decreased apoptosis, elevated DNA repair mechanisms, and altered drug transport mechanisms) against chemotherapy drugs. Recently, Chao et al. (2026) identified a novel partial epithelial-mesenchymal transition (pEMT)-associated transcriptomic signature that effectively stratified patients with ovarian cancer according to prognosis [87]. Finally, chemoresistant ovarian cancer cell lines, with the mesenchymal phenotype, serve as strong evidence supporting the importance of EMT in the development of therapeutic resistance [88].

### 4.5. Maintenance of Cancer Stem Cell Phenotypes

The importance of EGFR signaling in an individual’s response to chemotherapy is demonstrated by the EGFR role in preserving cancer stem cell (CSC) populations. Specifically, CSCs are defined as a small subset of a tumor that possesses self-renewal capabilities, the ability to initiate a tumor, and the ability to be resistant to standard chemotherapy regimens [89,90]. Therefore, the expression of genes regulating stemness and progression of CSCs in response to chemotherapeutic drugs is mediated through pathogenic signaling pathways, primarily EGFR, which promote CSC survival through the activation of Notch signaling, Wnt/β-catenin signaling, and Hedgehog signaling [91,92]. Interestingly, the development of an epithelial to mesenchymal transition (EMT) phenotype in an individual’s CSCs can be caused by the activation of the above-mentioned pathways. Consequently, individuals who exhibit an activated EMT phenotype will have a higher likelihood of expressing additional stem-like features, as evidenced by the increased expression of the cell surface markers ALDH1 or CD44 [32,93]. Collectively, CSCs are able to escape the effects of conventional chemotherapy because they possess several altered mechanisms that impart resistance to the apoptotic process. The well-established mechanisms that provide CSC populations with enhanced resistance to standard chemotherapy drugs include DNA repair, drug efflux, quiescent cell cycles, and resistance to apoptosis [32,35]. Therefore, through the acquisition of these classic mechanisms of chemoresistance, CSC populations, despite initial therapy, are accountable for both the recurrence and the development of metastasis in neoplastic diseases.

## 5. EGFR and the Tumor Microenvironment (TME)

Ovarian cancer development and resistance to chemotherapy have been found to significantly involve the development of the tumor microenvironment. Increasing evidence indicates that EGFR-mediated signaling interacts with the tumor microenvironment to facilitate adaptive responses that promote chemoresistance and disease progression [94]. EGFR also affects how the tumor cells communicate with neighboring stroma, immune cells and blood vessels during the formation of the tumor microenvironment and thus contributes to the establishment of a supportive environment for the continued growth of tumors within the microenvironment and during their escape from treatment. The interaction of multiple cellular systems (tumor and surrounding cells) and thus the establishment of chemoresistant phenotypes are promoted through multiple forms of EGFR-mediated information transfer [17,50].

### 5.1. Stromal Interactions and Paracrine Signaling

The presence of cancer-associated stromal cells (including CAFs, MSCs, and adipocytes) is mainly responsible for ovarian cancers developing over time. The stimulation of EGFR signaling increases the amount of paracrine communication between tumors and their associated stromal cells. Tumor-derived ligands activate EGFR on the stromal cell and stimulate the production of cytokines that promote tumor growth by the stromal cell [95,96]. Furthermore, EGF, transforming growth factor-α (TGF-α), and amphiregulin (EGFR ligands derived from the tumor) activate the EGFR of the stromal cell and subsequently stimulate its intracellular signaling pathway, resulting in the secretory activity of interleukin-6 (IL-6), hepatocyte growth factor (HGF), and CXCL12 (ligands for stromal cell response to IL-6, HGF, and CXCL12) by the stroma [97]. The production of IL-6, HGF, and CXCL12 by activated CAFs induces the activation of intracellular signaling pathways downstream of EGFR (PI3K/AKT and MAPK), thereby promoting cancer cell survival and proliferation. Moreover, the reciprocal signaling pathway between the tumor and the stroma creates an environment in which apoptotic processes are inhibited and the invasive behavior of cancer cells is enhanced. EGFR signaling is also purported to promote extracellular matrix (ECM) remodeling through the upregulation of matrix metalloproteinases (MMPs), resulting in the promotion of tumor metastasis [98]. In addition, the availability of an adipocyte-rich microenvironment (e.g., omentum) surrounding a tumor promotes tumor progression by promoting EGFR activity (lipid uptake and metabolic adaptation), thereby enhancing the resistance of cancer cells to stress induced by chemotherapy [99].

### 5.2. Immune Modulation and Immune Evasion

EGFR signal transduction greatly contributes to the establishment of the immunosuppressive TME. For instance, EGFR signaling increases the expression of programmed death ligand-1 (PD-L1) via activation of the PI3K/AKT and STAT3 pathways, leading to T-cell exhaustion and diminished anti-tumor immunity [100,101]. Furthermore, activation of the EGFR promotes the secretion of immunosuppressive cytokines (IL-10 and TGF-β) that expand regulatory T (Treg) cells and inhibit dendritic cell maturation [102]. Therefore, this contributes to the inhibition of effective immune surveillance and chemotherapy/immunotherapy resistance. In addition, EGFR signaling predisposes TAMs to become M2 macrophages. M2 macrophages secrete pro-tumorigenic factors (e.g., VEGF and arginase-1), further inhibiting cytotoxic T cells and enhancing tumor progression [33,34,103]. Finally, EGFR signaling downregulates the expression of major histocompatibility complexes (MHC), leading to decreased antigen presentation and immune evasion. Together, these mechanisms protect tumor cells from immune-mediated elimination by establishing an immunosuppressive microenvironment.

### 5.3. Hypoxia and Metabolic Reprogramming

The tumor microenvironment (TME) of ovarian cancer is characterized by hypoxic regions also linked to EGFR signaling. Activated EGFR signaling stabilizes hypoxia inducing factor-1α (HIF-1α) through PI3-kinase/Akt/mTOR pathways and promotes the transcription of multiple genes associated with glycolysis, angiogenesis and survival [104,105]. The TME has undergone a metabolic change to predominantly use aerobic glycolysis (Warburg effect), which causes an increase in glucose uptake and produces larger volumes of lactate, resulting in an acidified TME that will negatively affect immune cells [106]. EGFR signaling also regulates lipid metabolism and glutamine utilization, thereby supporting biosynthesis and redox homeostasis. Higher concentrations of glutathione are responsible for the neutralization of reactive oxygen species (ROS) and are considered to be an important factor in the development of chemoresistance against platinum-based chemotherapy [107]. Hypoxia and EGFR signaling will also act synergistically to induce epithelial-mesenchymal transition (EMT), promoting increased plasticity, invasiveness, and stemness of tumor cells [108]. Furthermore, EGFR-mediated activation of transcription factors, Snail, Twist, and ZEB1, reinforces the process of EMT and the development of chemoresistance.

### 5.4. Angiogenesis and Metastatic Dissemination

The activation of EGFR signaling, a critical regulator for inducing angiogenesis, which is the basis for ovarian cancer, stimulates the expression of vascular endothelial growth factor (VEGF) via MAP kinase and hypoxia-inducible factor-1 (HIF-1) pathways, thereby enhancing the proliferation and formation of new capillaries in endothelial cells [109]. In addition, EGFR signaling facilitates the adhesion, invasion, and implantation of tumor cells onto peritoneal surfaces through the increased expression of adhesion molecules and matrix metalloproteinases (MMPs) [110]. In addition, EGFR facilitates the formation of pre-metastatic niches through tumor-derived exosomes, which contain EGFR ligands [111]. Importantly, EGFR signaling also contributes to the development of resistance to anti-angiogenic therapies. The presence of compensatory signaling routes and increased pericyte coverage can enable the maintenance of tumor blood vessels despite the presence of therapeutic agents [112].

## 6. Therapeutic Targeting of EGFR in Ovarian Cancer

**Figure 3:**
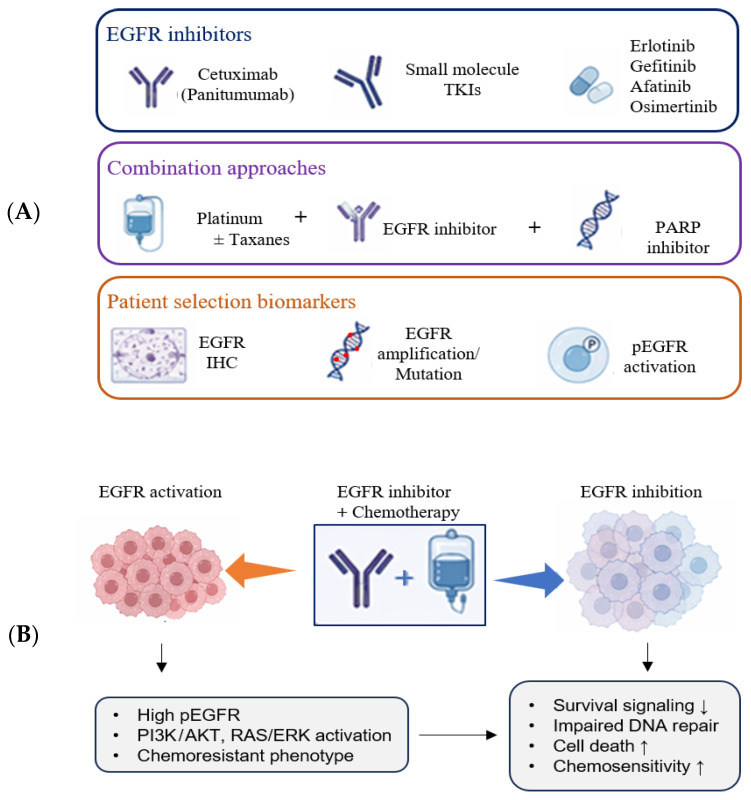
Targeting EGFR signaling to overcome chemoresistance in ovarian cancer. (**A**) Therapeutic strategies. EGFR-targeted therapies include monoclonal antibodies (e.g., cetuximab, panitumumab) and small molecule tyrosine kinase inhibitors (TKIs) such as erlotinib, gefitinib, afatinib, and osimertinib. Combination approaches with platinum-taxane chemotherapy and/or PARP inhibitors are under investigation. Biomarker-driven patient selection, based on EGFR expression (IHC), gene amplification, activating mutations, or phosphorylated EGFR (pEGFR), may improve therapeutic outcomes. (**B**) EGFR activation promotes chemoresistant phenotypes through activation of PI3K/AKT and RAS/ERK signaling. Inhibition of EGFR, alone or in combination with chemotherapy, suppresses survival signaling, impairs DNA repair mechanisms, restores apoptotic responses, and enhances chemosensitivity, resulting in synergistic anti-tumor effects and delayed resistance. EGFR, epidermal growth factor receptor; PARP, poly (ADP-ribose) polymerase; IHC, immunohistochemistry; pEGFR, phosphorylated EGFR; PI3K, phosphoinositide 3-kinase; AKT, protein kinase B; RAS, rat sarcoma; ERK, extracellular signal-regulated kinase; TKIs, tyrosine kinase inhibitors. ↑—Upregulation; ↓—Downregulation.

Ovarian cancer has a high frequency of overexpression of the EGFR as well as a connection to a pattern of growth in a more aggressive way, a higher likelihood for poor outcome, and resistance to conventional chemotherapy, making it a reasonable strategy for the use of anti-EGFR therapies. The main types of anti-EGFR therapies are small molecular weight tyrosine kinase inhibitors (TKIs), monoclonal antibodies, and their use in combination therapy (Figure 3A,B); however, there has been limited clinical success due to both innate and acquired resistance mechanisms to that therapy, and this fact calls for a better understanding of these mechanisms as well as new approaches to treating patients with ovarian cancer through EGFR-directed therapies.

### 6.1. Small Molecule Tyrosine Kinase Inhibitors (TKIs)

Despite the promising results of the preclinical studies, there has been little clinical efficacy of TKIs for the treatment of ovarian cancer. For example, erlotinib produced very low response rates and had no significant effect on progression-free survival in patients with platinum-resistant ovarian cancer [59,113]. In addition, the findings from two large clinical studies showed no evidence of survival benefit from the use of a TKI, regardless of whether the TKI was used as a single agent or in combination with a chemotherapy agent [114]. One of the major limitations to the efficacy of TKIs for the treatment of ovarian cancer is the lack of predictive biomarkers. As compared with non-small cell lung cancer (NSCLC), where the TKI treatment is initiated based on the presence of activating EGFR mutations, the presence of activating mutations in ovarian cancer is very rare and, subsequently, greatly reduces the likelihood that TKIs will have a therapeutic benefit to those patients who receive them [114,115]. Despite the promising results of the preclinical studies, there have been little clinical efficacy of TKIs for the treatment of ovarian cancer.

### 6.2. Monoclonal Antibodies Targeting EGFR

Monoclonal antibodies against EGFR that bind to the extra-cellular domain of the receptor (cetuximab, panitumumab, nimotuzumab and necitumumab) can inhibit ligand binding and receptor dimerization, preventing downstream signaling and triggering ADCC (antibody-dependent cellular cytotoxicity) to enhance immune-mediated apoptosis of malignant tumor cells [58]. Cetuximab has shown anti-proliferative and pro-apoptotic activity against ovarian cancer in preclinical (animal) models, and can sometimes reduce the development of resistance to chemotherapy when combined with chemotherapy [114]. However, cetuximab and other EGFR-targeting monoclonal antibodies have had little clinical efficacy in unselected populations of patients with ovarian cancer, whether used alone or in combination with other drugs [114]. The poor clinical outcomes associated with monoclonal antibodies targeting EGFR may be due to intratumoral heterogeneity, the presence of compensatory signaling pathways, and intrinsic resistance mechanisms. The lack of effective patient-selection strategies also contributes to the poor clinical outcomes associated with EGFR-targeting monoclonal antibodies.

### 6.3. Combination Strategies with Chemotherapy and PARP Inhibitors

The combined use of therapeutic agents has emerged as a promising strategy to overcome the limitations associated with single-agent therapies targeting EGFR. To enhance the cytotoxic properties of EGFR inhibitors when used together with chemotherapy, there are efforts to develop combination treatments to counteract any resistance mechanisms that may be present. Preclinical data show that combining EGFR TKIs and mAbs with chemotherapy may be viable for improving the sensitivity of ovarian cancer cells to both platinum-based and taxane chemotherapy by inhibiting survival pathways and enhancing apoptosis [114]. An example of this would be the synergy seen when adding gefitinib to an existing cytotoxic regimen. Additionally, combining EGFR-targeted agents with anti-angiogenesis agents (i.e., bevacizumab) or immune-based therapies could lead to superior clinical outcomes through the targeting of multiple tumor biology characteristics [116].

EGFR inhibitors in combination with PARP inhibitors represent another area of interest in terms of combination approaches. As EGFR signaling is shown to play a role in the DNA damage repair pathways, by inhibiting this pathway, there is the potential for an enhanced efficacy of the PARP inhibitors by further promoting the genomic instability and synthetic lethality of a given tumor [117]. Even though additional clinical trial data are forthcoming, this approach may permit an opportunity for researchers to target malignancies that are resistant to therapy using a rational approach that is targeted toward improving chemotherapy. Nevertheless, ongoing clinical development is plagued by overlapping toxicities, lack of predictive biomarkers, and new resistance mechanisms.

### 6.4. Mechanisms of Resistance to EGFR-Targeted Therapies

Ovarian cancer is resistant to EGFR treatments because of intrinsic and acquired mechanisms. Among these mechanisms, there are numerous factors that prevent EGFR drugs from binding to their target prior to the interaction occurring.

#### 6.4.1. Pre-Target Mechanisms

Genetic alterations (e.g., KRAS mutations) or the influence of other tumor microenvironmental factors can prevent effective binding of EGFR anti-tumor agents through competition for receptor sites (i.e., blocking, steric inhibition/occlusion) [58].

#### 6.4.2. On-Target Mechanisms

Furthermore, structural changes and mutations or deletions in the extracellular portion of the receptor (EGFR) impair the binding of antibodies and reduce their effect as anti-tumor agents [58]. In addition, overproduction of ligands may occlude or saturate receptor binding sites, thereby preventing antibody-mediated inhibition.

#### 6.4.3. Post-Target Mechanisms

Additionally, downstream pathways can signal in a manner that is independent of the EGFR, creating resistance to the anti-tumor effects of targeting the growth-related effects of EGFR. The continued activation of the primary downstream PI3K/AKT or MAPK pathway has been noted as a contributing factor to the development of drug resistance [58].

#### 6.4.4. Off-Target Mechanisms

Furthermore, the compensatory activation of alternative receptor tyrosine kinases (e.g., HER2, HER3, MET, FGFR, and/or VEGFR) can bypass the EGFR-mediated tumor growth and provide an alternative proliferative signal [58]. This redundancy in the signaling pathway network is significantly associated with the development of resistance to anti-EGFR medications.

#### 6.4.5. Genetic and Epigenetic Alterations

Secondary mutations, such as secondary EGFR mutations and other alternative pathways (e.g., HER2, MET), often develop as acquired resistance to TKIs [59]. There are also other factors, including epigenetic mechanisms, and non-coding RNA molecules such as microRNA (MiRNA) and lncRNA that have been implicated as modulating the effectiveness of EGFR-targeted therapies in clinical practice.

#### 6.4.6. Tumor Microenvironment (TME) and Exosomes

In mediating resistant (to treatment) behaviors, the tumor microenvironment has an unavoidable task of delivering survival-encouraging signals and encouraging immune evasion behaviors. Exosomes between cells (of similar origin) are known to move resistance-associated molecules to other cells as a way to create greater complexity in treatment response [58].

#### 6.4.7. Cross-Resistance Between TKIs and mAbs

Resistance to one type/class of EGFR-targeted therapy often creates resistance to other types/classes of EGFR-targeted therapy as well. Data show that ovarian cancer cells that developed resistance (to TKIs) typically also have developed resistance (to monoclonal antibodies) further confirming that some of the mechanisms that allow for the development of resistant behaviors are shared [115].

## 7. Emerging Strategies to Overcome EGFR-Driven Resistance

Oncogenic EGFR signaling in ovarian cancer has led to the development of chemotherapy-resistant tumors, which involve multiple factors that contribute to ovarian cancer treatment failure. The increase in tumor cell survival/proliferation through survival pathway activation, remodeling of tumor microenvironment (TME), and decreased immune detection are all significant contributors to chemoresistance. Recent advances are focused on combining targeted therapies with new delivery methods and combination strategies as the basis for targeting EGFR-mediated mechanisms of chemoresistance. This section will discuss new strategies that target EGFR-mediated resistance mechanisms.

### 7.1. Nanotechnology-Based Drug Delivery Systems

Nanotechnology is a promising new way for overcoming EGFR-related drug resistance in cancers through improved drug bioavailability, tumor targeting, and therapeutic effect. Conventional chemotherapy often has poor pharmacokinetics and increases in non-target toxicity that reduce the efficacy of these treatments for resistant tumors. Using nanocarriers (liposomes, polymeric nanoparticles, dendrimers, and inorganic nanomaterials) allows for controlled and targeted delivery of drugs to cancerous cells [118,119,120,121]. Nanoparticles can be designed to come into direct contact with the cancer cells that overexpress EGFR using ligand–receptor interactions. By contrast, folate receptor (α) and EGFR-targeted nanocarriers both allow for more effective accumulation of intracellular drugs, while decreasing the incidence of drug-related systemic toxicity [122]. These systems allow for the protection of the therapeutic agents against degradation, and therefore provide a means to bypass drug efflux and resistance mechanisms by providing a prolonged release of the therapeutic agent [123].

Also, by utilizing nanotechnology, co-delivery of multiple agents is feasible; therefore, multiple agent therapies such as chemotherapeutics combined with EGFR inhibitors and PARP inhibitors can be used in a single patient. Additionally, these combination therapies, when delivered together, allow for an increased level of synergy and allow for improved inhibition of compensatory signaling pathways [122]. Recent development of “stimuli-responsive” nanoparticles (e.g., pH-responsive, redox-responsive, or enzyme-responsive) allows for the development of nanoparticles that will release the medications delivered to the tumor microenvironment specifically at the tumor microenvironment. By allowing for this unique targeting, there will be decreased normal tissue toxicity associated with these therapies as well as improved clinical outcomes. Exploring nanoplatforms to deliver RNA-based therapeutics utilizing the delivery of siRNA and miRNA to block EGFR or downstream genes involved in resistance development is currently being evaluated [124,125]. Nanotechnology also enhances immunotherapy by allowing for direct delivery of immune checkpoint inhibitors to a tumor by activating an immune response and minimizing adverse side effects [126]. In addition to these advances, remaining challenges related to biological barriers, immune clearance, and scalability of these therapies are still not well understood. However, continued advancements of nanomedicine will offer tremendous opportunities to overcome EGFR-mediated resistance.

### 7.2. Dual and Multi-Targeted Inhibitors

EGFR signaling is rarely done alone but works rather in combination with numerous other carcinogenesis pathways, including VEGF (vascular endothelial growth factor) signaling, PI3K/AKT/mTOR, and MAPK/ERK. Blocking EGFR alone creates redundancy, leading to treatment resistance, thus multi-targeted and dual-inhibitor treatments are considered more effective forms of therapy [127]. Dual inhibitors directly inhibit EGFR and parallel pathways by using EGFR and VEGFR or EGFR and HER2 inhibitors which limit the nature of compensatory signaling loops that contribute to the survival of the tumor [128,129]. Multi-kinase inhibitors broaden this concept by blocking more than one type of tyrosine kinase involved in the tumor’s proliferation, angiogenesis, and metastasis [130,131].

EGFR inhibitors, when combined with PARP inhibitors, anti-angiogenic agents, and/or chemotherapeutics have shown benefits. Combination therapy exploits synthetic lethality and blocks several mechanisms behind treatment resistance, including enhanced DNA repair and tumor vascularization [93]. Another means of potentially disrupting the development of relapsed and resistant tumors utilizes CSC (cancer stem cell) treatment strategies. Multi-targeted approaches can inhibit both EGFR signaling and CSC-linked pathways such as the Wnt/β-catenin pathway and Notch pathway, thereby reducing the recurrence of tumors [91,92]. There may, however, be some challenges due to toxicity and the need for the precise identification of patients prior to treatment. Therefore, performing biomarker-driven therapy provides a basis for identifying patients most likely to benefit from multi-targeted therapies.

### 7.3. Epigenetic and Transcriptional Targeting

Epigenetic changes have a large impact on EGFR-driven chemoresistance by regulating gene expression through processes other than changes to the DNA sequence itself, such as DNA methylation, modification of histones, and regulating non-coding RNA (ncRNA). All of these factors together promote the survival of cancer cells, make them resistant to drugs, and enable escape from the immune system [5,31]. There are several epigenetic drugs that can reverse the aberrant epigenetic modifications and restore chemosensitivity, including inhibitors of DNA methyltransferases (DNMTs) and histone deacetylases (HDACs). For example, HDAC inhibition can inhibit downstream EGFR signaling and increase apoptosis in chemoresistant ovarian cancer cells [132,133,134].

Additionally, modulation of epigenetics has an effect on metabolic reprogramming and TME interactions. Histone modifications can increase glycolytic metabolism and contribute to resistance to targeted therapies, which highlights the importance of targeting epigenetic regulators with EGFR inhibitors [135]. Non-coding RNAs such as microRNAs and long non-coding RNAs are being identified as critical regulators of EGFR signaling. Developing targeted therapies that regulate these molecules may provide a new mechanism for overcoming EGFR-induced chemoresistance. Integrating epigenetic therapies with nanotechnology and immunotherapy could significantly improve the efficacy of treatment by reprogramming the tumor cells to make them sensitive to other treatment modalities.

### 7.4. Immunotherapeutic Approaches and Combination Regimens

Immunotherapy’s impact on cancer care has been profound, and combining approaches that target the EGFR with immunotherapy presents new ways to counteract chemoresistance. Ovarian cancers typically lead to an immunosuppressive tumor microenvironment that compromises the effectiveness of immune checkpoint inhibitors (ICIs). EGFR signaling promotes immune evasion by upregulating the expression of PD-L1 (phosphorylation of PD-L1) and suppressing the development of effective anti-tumor immune responses. By blocking PD-L1 using ICIs such as anti-PD-1/PD-L1 antibodies, you can restore the effectiveness of the immune system. The association of the PD-L1 pathway with chemoresistance makes PD-L1 a viable target for therapy [126].

Combination therapies are particularly encouraging in achieving these goals, including the use of EGFR inhibitors in combination with ICIs, chemotherapy in conjunction with immunotherapy, and PARP inhibitors with ICIs [136]. These combinations increase the immunogenicity of tumors, improve T-cell infiltration, and help overcome resistance mechanisms. Nanotechnology will also help to facilitate these therapies in addition to allowing for the targeted delivery of immunologic agents. Adoptive cell therapies, including the use of CAR-T cells that target antigen(s) associated with ovarian cancer such as mesothelin, are also currently being studied [137,138,139]. These are expected to target and kill tumor cells while avoiding traditional mechanisms of resistance. Another new avenue of research is the modulation of the tumor microenvironment with a focus on immune-suppressive cells such as regulatory T cells and M2 macrophages and the use of cytokines, which are expected to work synergistically with immunotherapy.

## 8. Clinical Evidence and Ongoing Trials

The clinical translation of targeting EGFR in ovarian cancer has been extensively investigated over the past two decades. Despite strong preclinical rationale linking oncogenic EGFR signaling to tumor proliferation, survival, and chemoresistance, clinical outcomes have been modest. This section critically evaluates completed clinical trials, highlights emerging therapeutic strategies, and discusses key barriers to successful clinical application.

### 8.1. Completed Clinical Trials Targeting EGFR

To date, only a limited number of early-phase clinical trials have investigated the clinical utility of these agents for the treatment of ovarian cancer. The investigational agents, which primarily included small molecule TKIs, specifically gefitinib and erlotinib, and monoclonal antibodies including cetuximab and trastuzumab, targeting EGFR, were used for the majority of clinical studies to date; however, the vast majority of the patients enrolled into these studies had platinum-resistant or recurrent ovarian cancer [113,140,141]. For gefitinib, the overall clinical efficacy as a single agent in the Phase II clinical trial settings was poor. Only a small percentage of patients had stable disease for a limited amount of time, while there were no patients who achieved a complete response [142,143,144,145]. Similarly, erlotinib was reported to demonstrate a low response rate of up to approximately 6% amongst heavily pre-treated patient populations. Likewise, monoclonal antibodies that also target the EGFR or HER2 have shown limited activity (< 10% response rate) and an overall short progression-free survival rate for this group of investigational agents [20,144,146,147].

Combination approaches have also been explored to improve their therapeutic effect when used alone. For example, combining EGFR inhibitors with chemotherapeutic or hormonal agents (due to its low toxicity, e.g., use of gefitinib + tamoxifen) provided only modest benefits with increased toxicity [51,144,147,148]. Using dual inhibitors to target multiple families of EGFR, such as the dual EGFR inhibitor, lapatinib, was associated with partial responses during early-phase studies but was not clinically applicable due to severe adverse events.

With many trials showing a low response rate of 0–6% for unselected ovarian cancer, the data suggest that using EGFR inhibitors as monotherapy will result in poor patient outcomes due to a lack of appropriate stratification [4]. Furthermore, clinical trials investigating the use of EGFR inhibitors as maintenance therapy following first-line chemotherapy did not show an increase in overall survival for patients with ovarian cancer [149]. These findings highlight an important limitation of using EGFR inhibitors in the treatment of ovarian cancer. Ovarian cancer has a low frequency of EGFR-activating mutations (<1%), which results in a low likelihood of response to EGFR-targeted monotherapy in ovarian cancer compared with non-small cell lung cancer (NSCLC), which has a high frequency of these mutations and is predictive of response [150]. Completed clinical trials of EGFR-targeted therapies are summarized in Table 3.

### 8.2. Ongoing Studies and Novel Approach

New discoveries in molecular characterization and the continual development of different types of therapies have resulted in ongoing interest among researchers concerning the use of EGFR-targeted therapies in treating ovarian cancer, even though early study results have produced only modest success rates. At this time, much of the research being conducted is centered on precision medicine and combination therapies. As we discussed earlier, there are currently clinical trials that will evaluate combining drugs that inhibit EGFR with ones that inhibit other similar signaling pathways, such as those seen with PI3K/AKT/mTOR inhibitors, MEK inhibitors, or PARP inhibitors, to understand the biological response by accounting for the compensatory pathways that will maintain resistance. For example, based on preclinical and early clinical data, using a combination of EGFR inhibition with PI3K inhibition could provide a synergistic effect for treating particular subsets of ovarian cancers [36].

In addition, the use of newer generation EGFR (inhibitors, such as specific mutant selective agents and irreversible TKIs) are being studied in clinical trials. For example, a case has been published of an individual with ovarian cancer who had an EGFR mutation and achieved an extended response to the TKI osimertinib; this demonstrates that there is potential for using target-directed therapeutics for molecularly defined patient subgroups [150]. While not exactly common occurrences, these types of cases serve as a proof-of-concept for the use of a precision medicine approach to cancer treatment. Within the clinical trial registries, there continues to be an increasing number of ongoing clinical research studies using EGFR targeting strategies as part of larger combinatory approaches to treatment. An example of this includes the study of combining selumetinib (a MEK-inhibitor) and PARP inhibitors for the treatment of RAS-pathway mutated ovarian cancers where there is a focus on designing therapies around the underlying pathways.

Another area of hope includes antibody-drug conjugates (ADCs) and bispecific antibodies directed against EGFR or other related receptors. These approaches aim to improve the tumor specificity of drugs and thereby improve the delivery of cytotoxic agents to tumors while reducing the systemic toxicity associated with many cancer therapies. Given the role that EGFR signaling plays in modulating the immune environment within the tumor and facilitating the immune evasion of the tumor, there is now interest in combining immunotherapy with EGFR-targeted therapies.

Biomarker-based trials are also growing in number, as there is an increased effort to identify predictive markers such as activation of downstream pathways, overexpression of ligands, gene amplification or EGFR mutations. In the coming years, the shift from a focus on therapy through biomarker identification toward a molecularly stratified approach will ultimately improve clinical outcomes for patients who receive EGFR-targeted therapy.

### 8.3. Clinical Translation Challenges

At present, there are multiple barriers that block success in clinically translating EGF receptor (EGFR)-targeted treatments for ovarian cancer.

#### 8.3.1. Predictive Biomarkers

One of the largest barriers to determining which patients will respond to EGFR inhibition is the lack of stable predictive biomarkers. For example, although many ovarian cancers overexpress EGFR, this does not mean that the patient will benefit from the treatment, either by having a clinical benefit or an increase in overall survival [4]. Conversely, EGFR mutations serve as strong predictors of prognosis in patients with lung cancer.

#### 8.3.2. Tumor Heterogeneity

Ovarian cancer is very heterogeneous, with a wide range of histopathological and molecular subtypes, each of which is variably reliant upon EGFR signaling; therefore, standardized treatment options are less effective. The existence of intratumoral heterogeneity creates further difficulties with treatment, as sub clonal populations may have variable sensitivity to EGFR inhibitors.

#### 8.3.3. Redundant/Compensatory Signaling

The tumor can evade suppression of the EGFR and continue to survive through activation of other pathways, such as PI3K/AKT, MAPK and hepatocyte growth factor receptor (heregulin) (MET). This redundancy contributes to intrinsic and acquired resistance to EGFR inhibition. The activity of these compensatory pathways has been demonstrated in preclinical studies to lead to a lack of efficacy of EGFR-targeted therapy when used as a single agent [36].

#### 8.3.4. Low Frequency of Mutation

EGFR gene mutations that activate it have a very low frequency (<1%) in ovarian cancer and therefore are not likely to be a major cause of malignancy as they are in many other tumors driven primarily by EGFR mutations. Almost all patients are therefore not expected to have the molecular dependence that is necessary for a successful response to EGFR-targeted therapies [150].

#### 8.3.5. Limitations of Clinical Trial Design

Although some potential response benefits can be realized from studies focusing on defined responsive subgroups, the results of much of the earlier data on EGFR-targeted therapies are limited by the fact that many early studies were performed in unselected patient populations. Additionally, biomarkers such as overall survival may not reflect the true benefit from targeted therapies in very small defined populations.

#### 8.3.6. Toxicity and Tolerance of Therapy

While combination therapies may offer a greater likelihood of producing greater anti-tumor effects, they also typically have a higher rate of toxicity, which leads to a greater number of people having their doses adjusted down or stopping therapy. Therefore, it is necessary to limit the application of multi-targeted approaches in the clinical environment.

#### 8.3.7. Adaptive Resistance Mechanism

By undergoing processes such as receptor mutation, activation of downstream signaling pathways, and through epithelial-mesenchymal transition (EMT), cancer cells can adapt dynamically to suppression of the EGFR. These mechanisms of adaptation are primarily responsible for failure of therapy and progression.

## 9. Future Perspectives

The role of EGFR signaling in the chemoresistance of ovarian cancer has opened up multiple avenues for future research and therapeutic innovation. EGFR-targeted strategies remain challenging to translate into durable clinical benefit due to tumor heterogeneity, adaptive resistance mechanisms, and pathway redundancy. Therefore, developing proactive precision-based therapy strategies improve clinical outcomes for patients with ovarian cancer. Here, we discussed key future perspectives on EGFR signaling in the chemoresistance of ovarian cancer.

### 9.1. Biomarker-Driven Patient Stratification

Future research should focus on determining predictive biomarkers of activation in the EGFR signaling pathway. Molecular markers such as EGFR gene mutations, amplification status, downstream signal transduction elements (backwards to downstream targets of EGFR which ultimately include genes such as PIK3CA and KRAS), as well as profiling circulating tumor DNA, will facilitate tailoring the treatments of individual patients based on their predicted response to EGFR-targeted therapy. This would also reduce the potential adverse events from therapies that would not benefit a patient.

### 9.2. Multi-Omics and Systems Biology Approaches

To fully investigate the complexity of resistance networks associated with EGFR, it is critical to integrate genomics, transcriptomics, proteomics, and metabolomics into multi-omics profiling efforts. Evidence generated through multi-omics profiling can provide information about new regulatory nodes, feedback loops, and compensatory pathways that contribute to drug resistance. Systems biology tools with artificial intelligence and computer modeling will assist researchers in creating predictive models of treatment response and resistance evolution leading to a better overall understanding of a tumor’s behavior.

### 9.3. Precision Oncology and Personalized Medicine

Ovarian cancer clinical management is expected to change through developments in precision oncology. Treatment targeted at an individual patient’s genetic makeup, tumor heterogeneity, and microenvironment will lead to longer survival and fewer recurrent cases by using a combination of therapies such as EGFR inhibitors with chemotherapy, immunotherapy, or agents that cause ferroptosis will be able to circumvent the mechanisms of resistance. Use of measuring therapy responses in real time and creating flexible clinical trials will contribute toward the development of personalized therapies.

## 10. Conclusions

In ovarian cancer, oncogenic EGFR signaling is one of the major mechanisms of chemoresistance because it also promotes proliferation, inhibits apoptosis, repairs DNA damage, causes EMT, and sustains cancer stem cells through signaling pathways such as PI3K/AKT, MAPK/ERK and JAK/STAT. Collectively, these pathways enable tumor cells to evade the cytotoxicity produced by chemotherapy, thus continuing to spread the disease. EGFR signaling can serve as a prognostic biomarker and a treatment target because it has been clinically shown to be associated with low response to therapy, high recurrence rates and decreased overall survival. However, the lack of efficacy of EGFR-targeted therapies as monotherapy shows the complicated nature of the resistance networks and need for more effective strategies.

Future therapeutic strategies should focus on the use of combination therapies that co-target EGFR with either compensatory or downstream pathways, application of predictive biomarkers to classify patients and development of new inhibitors to counteract adaptive resistance. It is anticipated that advancements in the fields of systems biology and precision oncology will increase the efficacy of EGFR-targeted therapies and ultimately improve clinical outcomes for patients with ovarian cancer.

## Figures and Tables

**Table 1:** Major EGFR-mediated signaling pathways involved in chemoresistance in ovarian cancer.

Sl. No	EGFR-Associated Pathway	Mechanism Contributing to Chemoresistance	Major Downstream Effects	Therapeutic Implications	Ref.
1	EGFR/PI3K/AKT/mTOR pathway	Persistent EGFR activation stimulates PI3K/AKT signaling, promoting survival under chemotherapeutic stress	Inhibition of apoptosis, enhanced proliferation, platinum resistance, metabolic adaptation	PI3K, AKT, and mTOR inhibitors combined with platinum therapy	[5,27,28]
2	EGFR/RAS/RAF/MEK/ERK pathway	EGFR-induced MAPK activation enhances tumor cell proliferation and stress adaptation	Increased cell cycle progression and reduced chemosensitivity	MEK/ERK inhibitors as sensitizing agents	[30,31]
3	EGFR/STAT3 signaling	Constitutive STAT3 activation downstream of EGFR promotes inflammatory and survival signaling	Stemness maintenance, immune evasion, anti-apoptotic signaling	STAT3 inhibitors may reverse drug resistance	[5,32]
4	EGFR-mediated EMT signaling	EGFR activation induces EMT through transcription factors such as Snail and Twist	Enhanced invasion, metastasis, and multidrug resistance	EMT-targeted therapy combined with EGFR inhibition	[33,34]
5	EGFR-cancer stem cell (CSC) axis	EGFR signaling supports ovarian cancer stem cell maintenance and tumor recurrence	Self-renewal, tumor relapse, resistance to cisplatin and paclitaxel	CSC-targeted nano therapy and EGFR blockade	[32,35]

**Table 2:** Molecular mechanisms and their targets linked with EGFR signaling contribute to chemoresistance in ovarian cancer.

Sl.no.	Mechanisms	Role of EGFR Signaling	Representative Biomarkers/Targets	Consequence in Ovarian Cancer	Ref
1	Enhanced DNA repair	EGFR signaling activates DNA damage response proteins	BRCA1/2, PARP, ATM	Efficient repair of platinum-induced DNA lesions	[34,75]
2	Apoptosis evasion	EGFR activates AKT and BCL-2 family proteins	BCL-2, Survivin, Caspases	Reduced chemotherapy-induced cell death	[5,28]
3	Drug efflux transporter activation	EGFR signaling increases ATP-binding cassette transporters	ABCB1/P-gp, MRP1	Reduced intracellular accumulation of chemotherapeutic drugs	[30,35]
4	Tumor microenvironment remodeling	EGFR promotes inflammatory cytokines, hypoxia, and stromal activation	HIF-1α, VEGF, IL-6	Protection of tumor cells from chemotherapy	[33,34]
5	Epigenetic reprogramming	EGFR-associated signaling alters histone modification and DNA methylation	EZH2, DNMT1, HDACs	Acquisition of resistant phenotypes	[5,31]
6	Cancer stemness induction	EGFR signaling supports stem cell transcriptional programs	CD44, ALDH1, NANOG	Tumor recurrence and therapeutic failure	[32]

**Table 3:** Completed clinical trials of EGFR-targeted therapies in ovarian cancer.

Sl. No.	Trial/NCT Number	EGFR Mutation/Expression Status	Phase	Drug	Treatment Regimen	Ref.
1	NCT00071955	EGFR-positive or unselected recurrent ovarian cancer; activating EGFR mutation detected in one responder	Phase II	Gefitinib	Gefitinib monotherapy (500 mg/day)	[142]
2	Not specified	Refractory/recurrent epithelial ovarian cancer; EGFR pathway evaluated pharmacodynamically	Phase II	Gefitinib	Gefitinib monotherapy	[143,144]
3	Not specified	Refractory or resistant ovarian cancer; EGFR-positive disease	Phase II	Gefitinib + Tamoxifen	Combination therapy	[144,147]
4	NCT00063401	EGFR-positive advanced ovarian, fallopian tube, or primary peritoneal cancer	Phase II	Cetuximab	Cetuximab + Paclitaxel + Carboplatin	[151]
5	Not specified	Newly diagnosed stage III/IV ovarian cancer; EGFR-unselected	Phase II	Erlotinib	Erlotinib + Paclitaxel + Carboplatin	[20,144]
6	Not specified	Chemo-naive ovarian cancer	Phase Ib	Erlotinib	Erlotinib + Docetaxel + Carboplatin	[146]
7	EORTC 55041	Unselected ovarian cancer after first-line chemotherapy	Phase III	Erlotinib	Erlotinib maintenance therapy vs. Placebo	[144,147]
8	NCT00130520	Advanced recurrent ovarian cancer progressing after platinum/taxane therapy	Phase II	Erlotinib + Bevacizumab	Combination targeted therapy	[149]
9	NCT00126542	Recurrent/metastatic ovarian epithelial, fallopian tube, or primary peritoneal cancer	Phase II	Erlotinib + Bevacizumab	Combination therapy	[149]
10	GOG HER2 Trial	HER2/ERBB2-positive ovarian cancer	Phase II	Trastuzumab	Trastuzumab monotherapy	[147]

## Data Availability

No new data were created or analyzed in this study. Data sharing is not applicable to this article.

## Abbreviations

The following abbreviations are used in this manuscript:

ABCB1 (P-gp)	ATP-binding cassette subfamily B member 1 (P-glycoprotein)
ABCC1	ATP-binding cassette subfamily C member 1
AKT	Protein kinase B
ALDH1A1	Aldehyde dehydrogenase 1 family member A1
Bcl-2	B-cell lymphoma 2
Bcl-xL	B-cell lymphoma-extra large
BRCA1	Breast cancer gene 1
BRCA2	Breast cancer gene 2
c-MET	Cellular Mesenchymal-Epithelial Transition factor
c-Myc	Cellular myelocytomatosis oncogene
COX-2	Cyclooxygenase-2
CXCL12	C-X-C motif chemokine ligand 12
DNA	Deoxyribonucleic acid
DNA-PK	DNA-dependent protein kinase
ECMs	Extracellular matrix
EGFR	Epidermal growth factor receptor
EMT	Epithelial-mesenchymal transition
EOC	Epithelial ovarian cancer
ERK	Extracellular signal-regulated kinase
EV	Extracellular vesicles
HB-EGF	Heparin-binding EGF-like growth factor
HGSOC	high-grade serous ovarian cancer
HIF-1α	Hypoxia inducing factor-1α
IGF-1R	Insulin-like Growth Factor 1 Receptor
IHC	Immunohistochemistry
IL-6	Interleukin-6
IL-8	Interleukin-8
JAK	Janus kinase
MCL-1	Myeloid cell leukemia sequence 1
MEK	Mitogen-activated protein kinase
mRNA	messenger RNA
mTOR	Mammalian target of rapamycin
NF-Κb	Nuclear factor kappa B
OVC	Ovarian cancer
PARP	Poly (ADP-ribose) polymerase
pEGFR	Phosphorylated EGFR
PI3K	Phosphoinositide 3-kinase
PD-L1	Programmed death ligand-1
RAD51	RAD51 recombinase
RAF	Rapidly accelerated fibrosarcoma kinase
RAS	Rat sarcoma
RNA	Ribonucleic acid
ROC	Receiver Operator Characteristic
RTKs	Receptor tyrosine kinases
STAT	Signal transducer and activator of transcription
Survivin	Baculoviral IAP repeat containing protein 5 (BIRC5)
TCGA	The Cancer Genome Atlas
TGF-α	Transforming growth factor-α
TKIs	Tyrosine kinase inhibitors
TME	Tumor microenvironment
